# Elafibranor emerged as a potential chemotherapeutic drug for non-muscle invasive bladder cancer

**DOI:** 10.1016/j.cellin.2024.100149

**Published:** 2024-01-29

**Authors:** Wang Wang, Danni Shan, Guanyi Wang, Xiongmin Mao, Wenjie You, Xiaolong Wang, Zijian Wang

**Affiliations:** a Department of Urology, Cancer Precision Diagnosis and Treatment and Translational Medicine Hubei Engineering Research Center, Zhongnan Hospital of Wuhan University, Wuhan, 430071, China; b Orthopedic Hospital, The First Affiliated Hospital, Jiangxi Medical College, Nanchang University, Nanchang, 330006, China; c Lewis Katz School of Medicine, Temple University, Philadelphia, PA, 19140, USA; d Department of Biomedical Engineering and Hubei Province Key Laboratory of Allergy and Immune Related Disease, Taikang Medical School (School of Basic Medicine Sciences), Wuhan University, Wuhan, 430071, China

**Keywords:** Elafibranor, Bladder cancer, Conditional reprogramming cell, Drug development, Preclinical study

## Abstract

Intravesical infusion of chemotherapeutics is highly recommended by several clinical guidelines for treating nonmuscle invasive bladder cancer (NMIBC). However, cytotoxic chemotherapeutics can cause a series of side effects, which greatly limits their application. Herein, a starvation therapy strategy was proposed, and elafibranor (ELA) was validated as a safe chemotherapeutic for NMIBC. The results showed that 20 μM ELA was sufficient to inhibit the proliferation and migration of bladder cancer cells and increase the level of intracellular reactive oxygen species (ROS). Furthermore, 2 mg/kg ELA treatment blocked the growth of primary tumors in an immunodeficient model by inhibiting proliferation and inducing apoptosis and improved the survival time of immunocompetent model mice. ELA treatment up to 10 mg/kg met the general safety requirements. We also established a patient-derived conditional reprogramming cell (CRC) model to assess the clinical translational potential of ELA. The antitumor effect and antitumor specificity of ELA treatment were confirmed. This work not only identified a promising chemotherapeutic for NMIBC but also provided a potential methodological system for drug discovery.

## Highlights

• A strategy of starvation therapy is developed for non-muscle invasive bladder cancer.
• Elafibranor functions partially through PPAR-α/β to inhibit bladder cancer cells growth.
• Elafibranor with antitumor activity and biosafety has potential for clinical transformation.

## Graphical abstract

Intravesical infusion of Elafibranor for NMIBC treatment.

**Figure:**
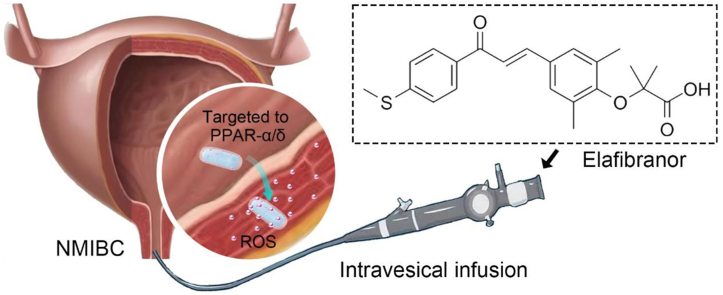

## 1 Introduction

Nonmuscle invasive bladder cancer (NMIBC) is a type of urological malignancy that occurs on the surface of the urothelium. Transurethral resection of bladder cancer (TURBC) is considered the gold standard for treating NMIBC, and the 5-year survival rate is approximately 90% (Flaig et al., 2020; Sanli et al., 2017). As an important supplement, postoperative intravesical infusion (IVI) is strongly recommended by several professional guidelines (Babjuk et al., 2019; Chang et al., 2016). IVI is conducive to eliminating residual tumor lesions and preventing local recurrence (Kamat et al., 2016). However, commonly used chemotherapeutics, such as gemcitabine, valinomycin, and pirarubicin, have nonspecific cytotoxic effects and are likely to cause various side effect (Huang et al., 2021; Wei et al., 2023; Zhu et al., 2023). Thus, the screening of advanced chemotherapeutics is urgently needed.

In recent decades, starvation therapy (ST) has been proven to be a feasible strategy for tumor intervention (Ding et al., 2020; He et al., 2020). Compared with normal bladder epithelial cells, NMIBC cells take in more glucose to support their energy requirements (Wu et al., 2021). Based on this characteristic, fludeoxyglucose F18 (18F-FDG) has been applied to visualize primary and metastatic NMIBC via positron emission tomography (PET) (Witjes et al., 2021; Civelek et al., 2021). Lipid metabolism is another energy source for NMIBC cells (Yang et al., 2022). In previous reports, our group revealed the enhanced lipid metabolism in bladder cancer and screened several anti-lipid gene targets and drugs (Cheng et al., 2019a, 2019b; Wang et al., 2016). It is assumed that blocking glucose and lipid metabolism at the same time will effectively reduce the energy supply of NMIBC cells and achieve suitable antitumor effects.

Elafibranor (ELA), also named GFT505, was first reported in 2010 (Hanf et al., 2010). ELA is the first coagonist of peroxisome proliferator-activated receptor-alpha and -delta (PPAR-α/δ) (Li et al., 2021). In the past ten years, the use of ELA for the treatment of obesity-related diseases, such as nonalcoholic steatohepatitis (NASH), dyslipidemia, and type 2 diabetes has been widely investigated (Cariou & Staels, 2014; Gross et al., 2017). Notably, an important milestone of ELA as a drug is its approval for primary biliary cholangitis by U.S. Food and Drug Administration (FDA) in 2017. The underlying mechanisms of ELA treatment have been partially characterized. Cariou et al. reported that ELA possesses dual hypoglycemic and hypolipidemic effects (Cariou et al., 2011). Based on this dual energy blocking strategy, ELA has emerged as a promising candidate for treating NMIBC.

A series of clinical trials of ELA have been carried out, but the results have led to inconsistent conclusions. In a phase II, multicenter, double-blinded, randomized controlled trial, ELA was administered to patients with NASH but not cirrhosis at a dose of 120 mg/d for 52 weeks. The results showed that the liver fibrosis stage, liver enzymatic activity, and lipid levels were significantly lower (Ratziu et al., 2016). Positive results also appeared in another phase II randomized placebo-controlled trial of primary biliary cholangitis (Schattenberg et al., 2021). However, the phase III clinical trial of NASH was abandoned because ELA treatment did not reach the primary endpoint of alleviating NASH without worsening liver fibrosis. Currently, the clinical translation potential of ELA remains unclear. In this context, the clinical efficacy and biosafety of ELA treatment should receive special attention.

High-quality preclinical studies play a vital role in the research and development (R&D) of new drugs (Chen et al., 2023; Wang et al., 2023a, 2023b; Zhao et al., 2023). Immortalized cell lines and tumor-bearing nude mice have traditionally been used for preclinical studies *in vitro* and *in vivo*. These research tools suffer from several shortcomings, including heterogeneity (Cassidy et al., 2015), and advanced research tools are urgently needed. Herein, we provided two substitutes. First, Liu's group developed a bladder cancer patient-derived conditional reprogramming cell (CRC) line (Jiang et al., 2019; Liu et al., 2017). The CRC line retains the characteristics of donor patients and thus has more advantages than immortalized cell lines. Second, tumor growth and tumor metastasis are strongly related to the body's immune system (Wang et al., 2021; Zhao & Cui, 2020). For most preclinical studies, immunocompetent animal models are superior to immunodeficient nude mouse models. It was reported that the immunocompetent bladder cancer model could be feasibly established using MB49 cell-bearing C57BL/6 mice (Mossberg et al., 2010; Yuen et al., 2018).

Herein, we hypothesized that ELA has application potential for the treatment of NMIBC. The clinical efficacy and biosafety of ELA treatment were investigated using a series of progressive and bench-to-bedside research tools, including immortalized cell lines, immunodeficient mice, immunocompetent mice and patient-derived CRC cells. The findings of this study provide important information for future clinical trials. Furthermore, our research tools and methods may open new avenues for drug discovery.

## 2 Results

### 2.1 ELA inhibited cell proliferation and migration and increased ROS levels

The proliferation and migration ability of ELA-treated bladder cancer cells and the levels of ROS were determined. ELA is a coagonist of PPAR-α/δ,; thus, bladder cancer cell lines with high PPAR-α/δ expression are more suitable for *in vitro* cell experiments. In the present study, the expression of PPAR-α/δ in four kinds of bladder cancer cells was measured via qRT‒PCR. The results are shown in Fig. S1. Compared to that in normal bladder epithelial cells (SV), PPAR-α/δ was highly expressed in UMUC3 cells and T24 cells but weakly expressed in RT4 cells and J82 cells. Thus, UMUC3 cells and T24 cells were selected for subsequent study.

**Fig. 1:**
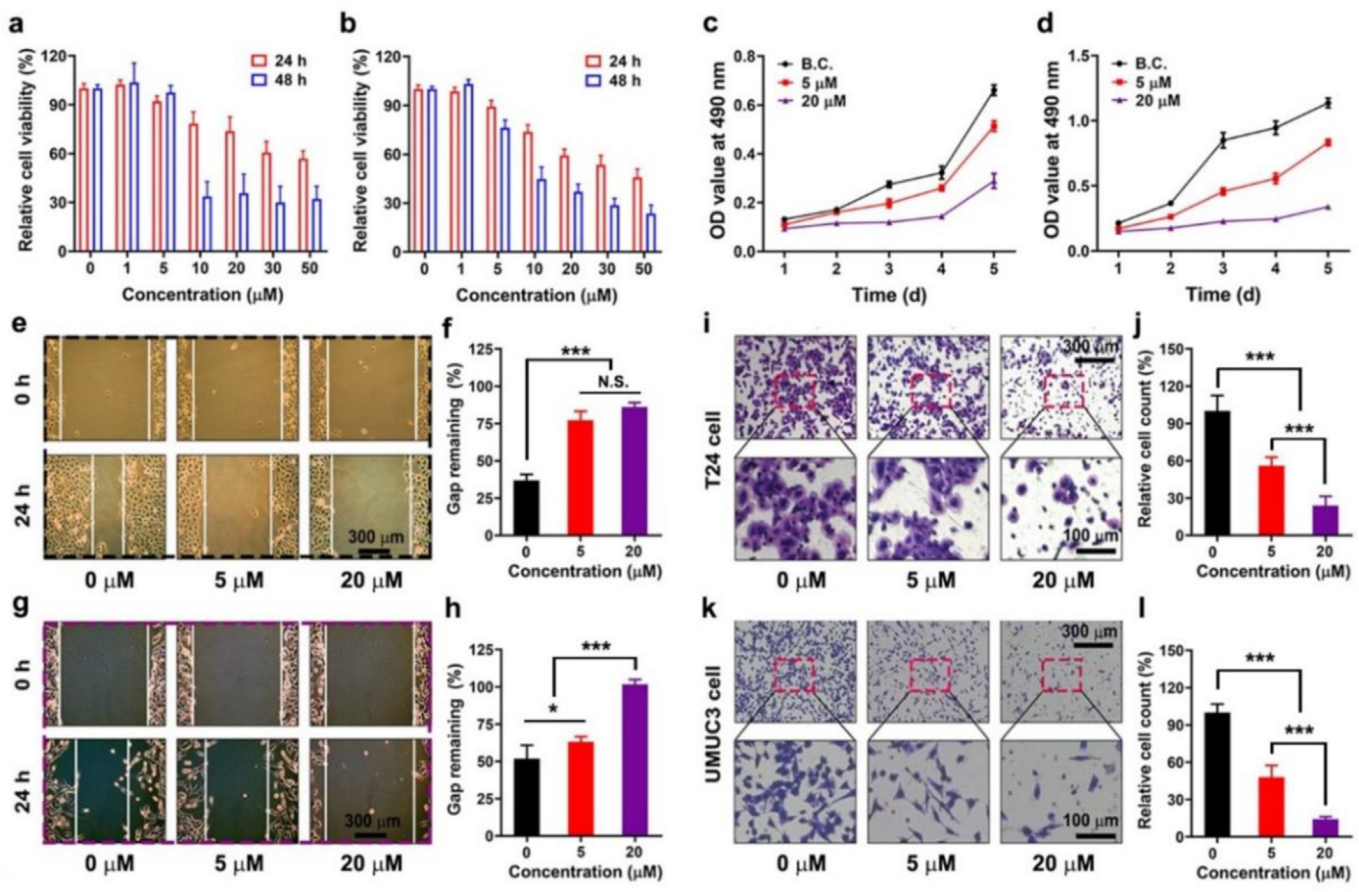
**ELA inhibited the viability and migration of bladder cancer cells.** (a) The relative viability of T24 cells treated with ELA in different concentration (n = 6); (b) Results of UMUC3 cells (n = 6); (c) The proliferation curves of T24 cells treated with 0 μM, 5 μM and 20 μM of ELA (n = 6); (d) Results of UMUC3 cells (n = 6); (e, f) Wound healing images and quantitative results of the T24 cell (n = 3). Scale bar: 300 μm; (g, h) Results of UMUC3 cell (n = 3). Scale bar: 300 μm; (i, j) Transwell images and quantitative analysis of T24 cell (n = 4). Scale bar: 100 and 300 μm; (k, l) Results of UMUC3 cell (n = 4). Scale bar: 100 and 300 μm. Values are expressed as the mean ​± ​SD, ∗∗*P* ​< ​0.01. ∗∗∗*P* < 0.001, N.S. indicated no significance.

The viability of the treated cells was evaluated by MTT assay. As shown in Fig. 1a, the relative viability of T24 cells decreased gradually with increasing ELA concentration. A similar trend was observed for UMUC3 cells, as shown in Fig. 1b. Based on these results, three independent groups, namely, a blank control group (0 μM), a low-dose group (5 μM) and a high-dose group (20 μM), were established according to the concentration of ELA. The inhibitory effect of ELA on proliferation was further verified after 5 days. Fig. 1c–d illustrates that ELA treatment inhibited tumor cell proliferation in a time- and dose-dependent manner. As shown in Fig. S2, the mRNA expression of PPAR-α/δ was downregulated by siRNA, which led to the recovery of cell proliferation in ELA-treated cells. These results suggested that the anti-proliferative effect of ELA might be attributed to PPARα/δ-mediated pathways.

As shown in Fig. 1e–f, the migration ability of ELA-treated T24 cells was assessed via a wound healing assay. After 24 h of incubation, the remaining wound gaps were 36.8% ± 4.1% for the 0 μM group, 77.2% ± 6.1% for the 5 μM group and 86.0% ± 3.0% for the 20 μM group. A significant difference was found between the 0 μM group and the other groups (*P* < 0.001). The effect of ELA on the migration ability of UMUC3 cells is shown in Fig. 1g–h; the migration ability was 51.9% ± 8.9% in the 0 μM group, 63.0% ± 3.6% in the 5 μM group and 101.7% ± 3.2% in the 20 μM group. A significant difference was found between the groups (*P* < 0.001). The migration ability of T24 and UMUC3 cells was effectively inhibited by ELA treatment.

The Transwell chamber assay is another method for evaluating cell migration ability. ELA-containing culture medium was added to the upper layer of the Transwell chamber. The cells migrated from the ELA-containing culture medium to the ELA-free culture medium. The results of T24 and UMUC3 cells are shown in Fig. 1i-l. The relative count of migrated T24 cell was 100.0% ± 12.5% for 0 μM group, 55.9% ± 7.0% for 5 μM group and 23.9% ± 7.5% for 20 μM group; and that of migrated UMUC3 cell was 100.0% ± 6.8% for 0 μM group, 47.9% ± 9.5% for 5 μM group and 14.4% ± 1.8% for 20 μM group. The groups exhibited significant differences (*P* < 0.001). These results were consistent with those of the wound healing assay.

Cell proliferation and migration play vital roles in the metastasis and recurrence of malignant tumors. In the present study, ELA exhibited dual anti-proliferative and anti-migratory effects in an immortalized cell model. Our results indicated that ELA could effectively inhibit the growth of primary tumors and block tumor cells from migrating into blood vessels and lymph nodes to form metastatic lesions.

**Fig. 2:**
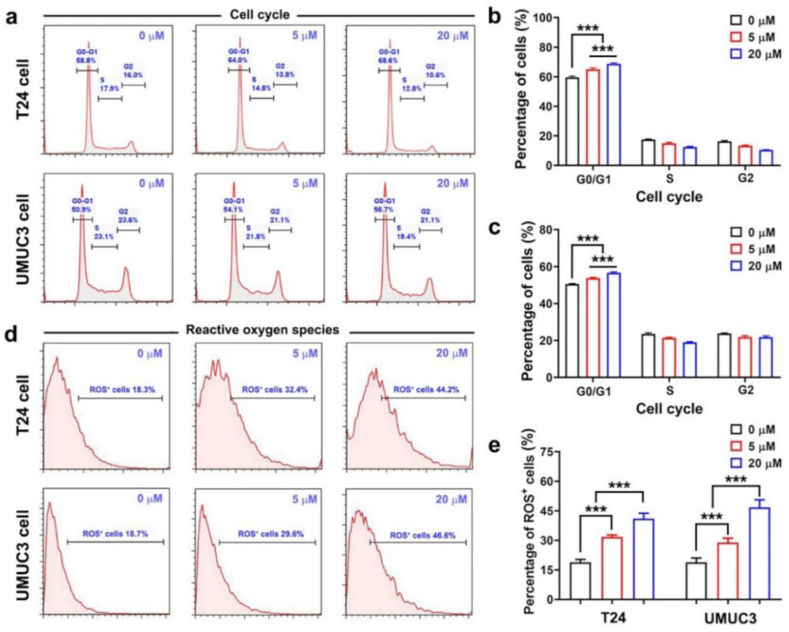
**ELA arrested cell cycle and upregulated the level of ROS.** (a) Cell cycle of T24 and UMUC3 cell was detected by flow cytometry; (b) Quantitative results of T24 ​cell (n ​= ​3); (c) Quantitative results of UMUC3 cell (n ​= ​3); (d) Reactive oxygen species (ROS) of T24 and UMUC3 cells was detected by flow cytometry; (e) Quantitative results of ROS positively stained cells (n ​= ​3). Values are expressed as the mean ​± ​SD, ∗∗∗*P* < 0.001.

Flow cytometry was performed to further investigate how ELA treatment affects the cell cycle. As shown in Fig. 2a–c, the cell cycle of ELA-treated bladder cancer cells was effectively arrested in the G0/G1 phase. The percentage of T24 cells in G0/G1 phase was 59.6% ± 0.7% after treatment with 0 μM ELA, 65.0% ± 1.1% with 5 μM ELA, and 68.8% ± 0.6% with 20 μM ELA; For UMUC3 cells, it was 50.5% ± 0.3% for 0 μM ELA, 53.7% ± 0.4% for 5 μM ELA and 56.6% ± 0.5% for 20 μM ELA. The groups exhibited significant differences (*P* < 0.001). The expression of cell cycle arrest markers, including PCNA and cyclin D1, was also detected via western blotting. As shown in Fig. S3, the protein expression of UMUC3 cells and T24 cells was downregulated by ELA treatment. Cell cycle regulation is strongly related to cell proliferation. In the present study, ELA increased the percentage of cells in the G0/G1 phase, thus inhibiting cell proliferation.

As shown in Fig. 2d–e, the intracellular ROS level was determined via flow cytometry. The percentage of ROS^+^ T24 cells increased from 18.8% ± 1.5% (0 μM group) to 31.8% ± 0.9% (5 μM group) and 41.0% ± 2.8% (20 μM group); in UMUC3 cells, the percentage increased from 18.8% ± 2.2% (0 μM group) to 28.9% ± 2.2% (5 μM group) and 46.7% ± 3.9% (20 μM group). A significant difference was observed between the groups (*P* < 0.001). ROS are a hallmark of tumorigenesis, and a series of metabolic pathways and metabolites are involved in ROS production. In the present study, intracellular catalase (CAT) activity and malondialdehyde (MDA) levels were determined via biochemical methods. As shown in Fig. S4, the level of MDA significantly decreased in response to ELA treatment, and the activity of the CAT enzyme significantly increased. As shown in Fig. S5, the apoptosis rate of UMUC3 and T24 cells was not influenced by ELA treatment. Moreover, N-acetylcysteine (NAC) could clear the ELA-induced ROS. As shown in Fig. S6, the cell proliferation of the ELA group and the ELA ​+ ​NAC group was not significantly different (*P* > 0.05), indicating that ROS and cell proliferation did not interact in this study. In conclusion, the antitumor activity of ELA might be independently attributed to cell cycle arrest and ROS generation.

### 2.2 ELA inhibited tumor progression and recurrence with suitable biosafety

**Fig. 3:**
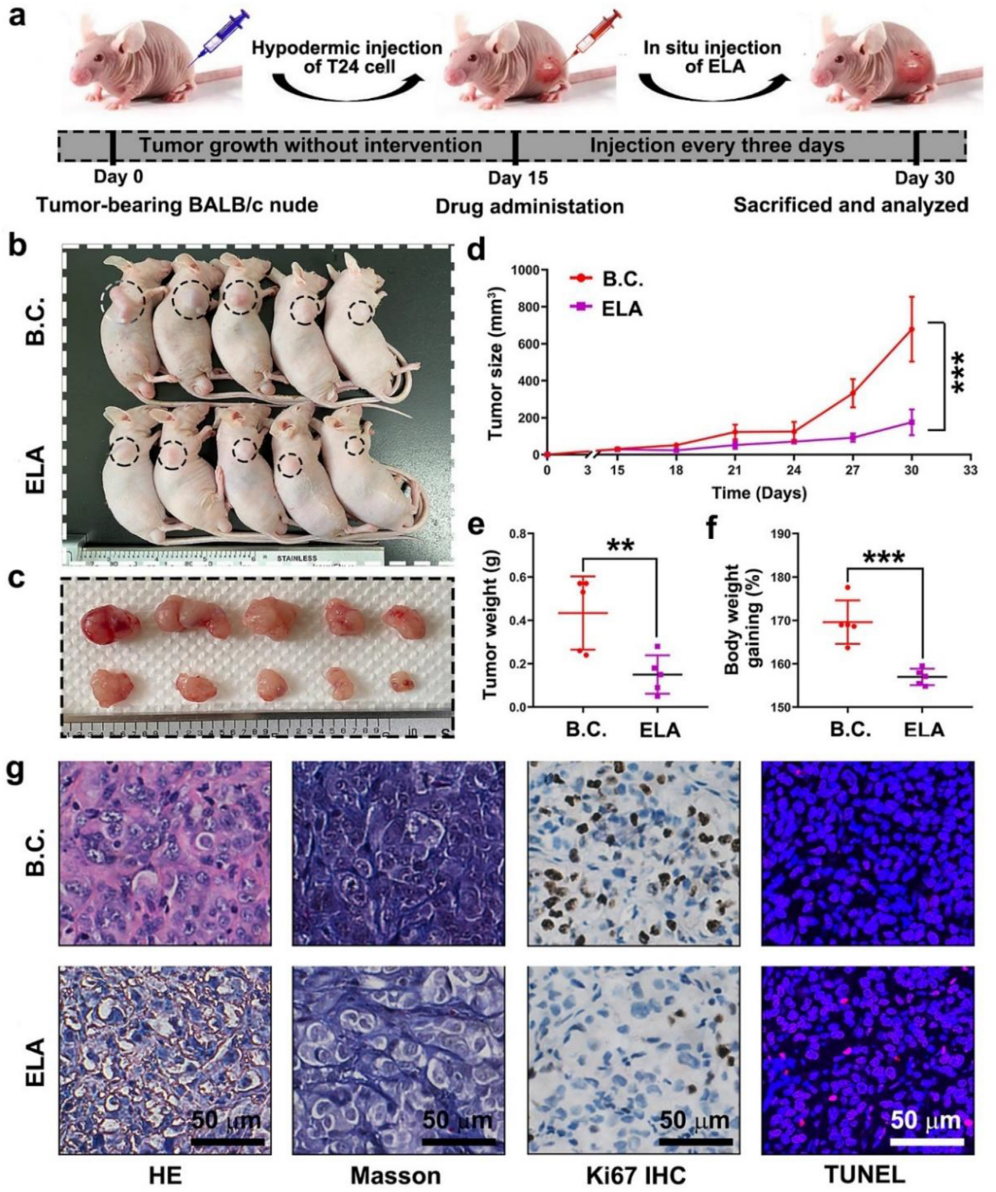
**ELA inhibited the progression in an immunodeficienttumor-bearingmodel.** (a) Schematic illustration of T24 cell-bearing BALB/c nude mice model and the drug administration plan; (b) The optical image of tumor-bearing mice at day 30. The black circle indicated neo-tumor tissue; (c) The optical image of neo-tumor tissue; (d) The growth curve of tumor size (n = 5); (e, f) Results of tumor weight and body weight gaining at day 30 (n = 5); (g) Histological images of neo-tumor tissue, including HE, Masson, Ki67 and TUNEL. Scale bar: 50 μm. Values are expressed as the mean ​± ​SD, ∗∗*P* ​< ​0.01, ∗∗∗*P* < 0.001.

The antitumor potential of ELA *in vivo* was evaluated using a traditional immunodeficient tumor-bearing model. This animal model is schematically illustrated in Fig. 3a. T24 cells were subcutaneously transplanted into BALB/c nude mice to allow for exogenous cell growth. After 15 days of feeding, the neotumors became visible to the naked eye. The ELA group was treated with ELA at a dose of 2 mg/kg, and the process was repeated every three days. The blank control (B.C.) group was treated with an equal amount of normal saline. The growth of the neotumors was monitored.

An optical image of the tumor-bearing nude mice is shown in Fig. 3b. The black circles indicate the neotumour sites. After the animals were sacrificed, all the neotumor tissue was resected. As shown in Fig. 3c, the tumors in the ELA group were obviously smaller than those in the B.C. group. The growth curve of the tumor size is shown in Fig. 3d. The tumor size increased incrementally over 15 days. On Day 30, the mean tumor size was 678.8 ± 175.9 mm^3^ in the B.C. group and 175.1 ± 69.6 mm^3^ in the ELA group. There was a significant difference (*P* < 0.001). As shown in Fig. 3e, the tumor weights were 0.43 ± 0.17 g for the B.C. group and 0.15 ± 0.09 g for the ELA group. The difference between the two groups was significant (*P* < 0.01). These results suggested that the *in vivo* antitumor effect of ELA was robust.

As an important part of biosafety evaluation, the body weights of tumor-bearing animals were measured. As shown in Fig. 3f, the body weight gain was 169.6% ± 4.9% in the B.C. group and 156.9% ± 1.9% in the ELA group. Significant differences were observed (*P* < 0.001). The weight gain of the animals came from the simultaneous growth of the body and tumor tissue. In this study, the growth of the tumor played a major role. ELA administration did not have obvious side effects on body weight.

The neotumour tissue was further characterized by histopathological analysis (Fig. 3g). According to the HE staining images, the ELA group exhibited less tumor heterogeneity than the B.C. group. Moreover, the cytoplasmic staining of the ELA group was obviously shallow. As shown in the Masson's trichrome staining images, the amount of extracellular matrix (ECM) was obviously reduced by ELA treatment. Ki67 is a protein marker of tumor proliferation, and TUNEL is a marker of cell apoptosis. The ELA group had fewer Ki67^+^ cells but more TUNEL-positive cells. Thus, ELA inhibited the tumorigenesis of bladder cancer *in vivo*, partly through proliferation inhibition and induction of cell apoptosis.

**Fig. 4:**
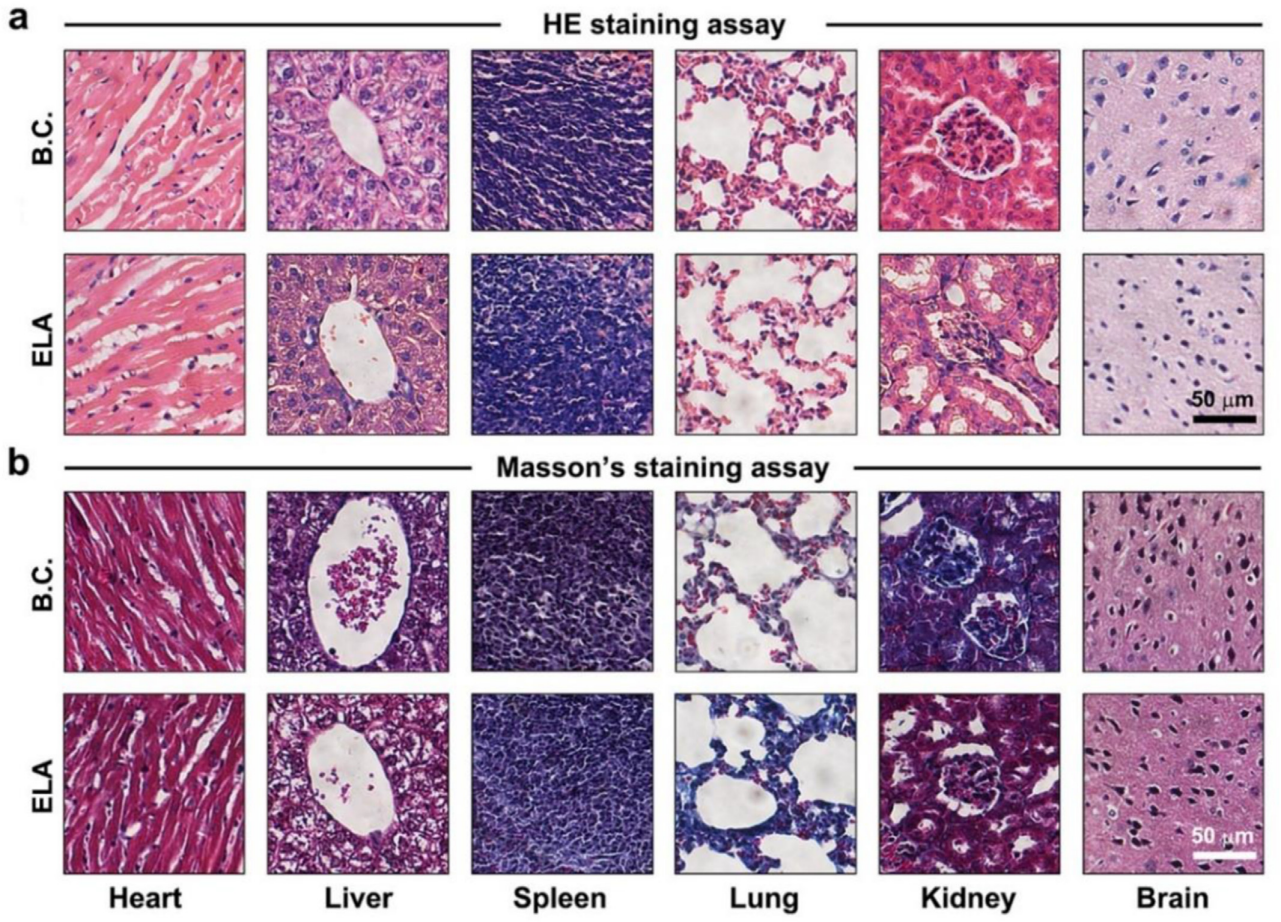
**ELA chemotherapy showed minimal organ toxicity.** (a) Images of organs stained with HE from BALB/C nude mice treated with ELA. The heart, liver, spleen, brain, lung and kidney were analyzed. Scale bar: 50 μm. (b) Masson's staining images. Scale bar: 50 μm.

As shown in Fig. 4a–b, HE and Masson's trichrome staining were performed to preliminarily evaluate the organ toxicity of ELA treatment. The tissue morphology of the heart, liver, spleen, lung and brain was normal, and the biosafety was suitable after 5 rounds of ELA administration at 2 mg/kg. In the ELA group, the kidney glomerulus exhibited mild atrophy, and the collagen content in the extracellular matrix (ECM) was obviously lower than that in the B.C. group. ELA is a dual PPARα/δ agonist that is mainly excreted through the kidney. Long-term, high-dose administration of ELA could increase the burden on the kidney. In the present study, these mild pathological changes did not directly lead to renal injury. However, the potential nephrotoxicity of these agents should be given close attention.

**Fig. 5:**
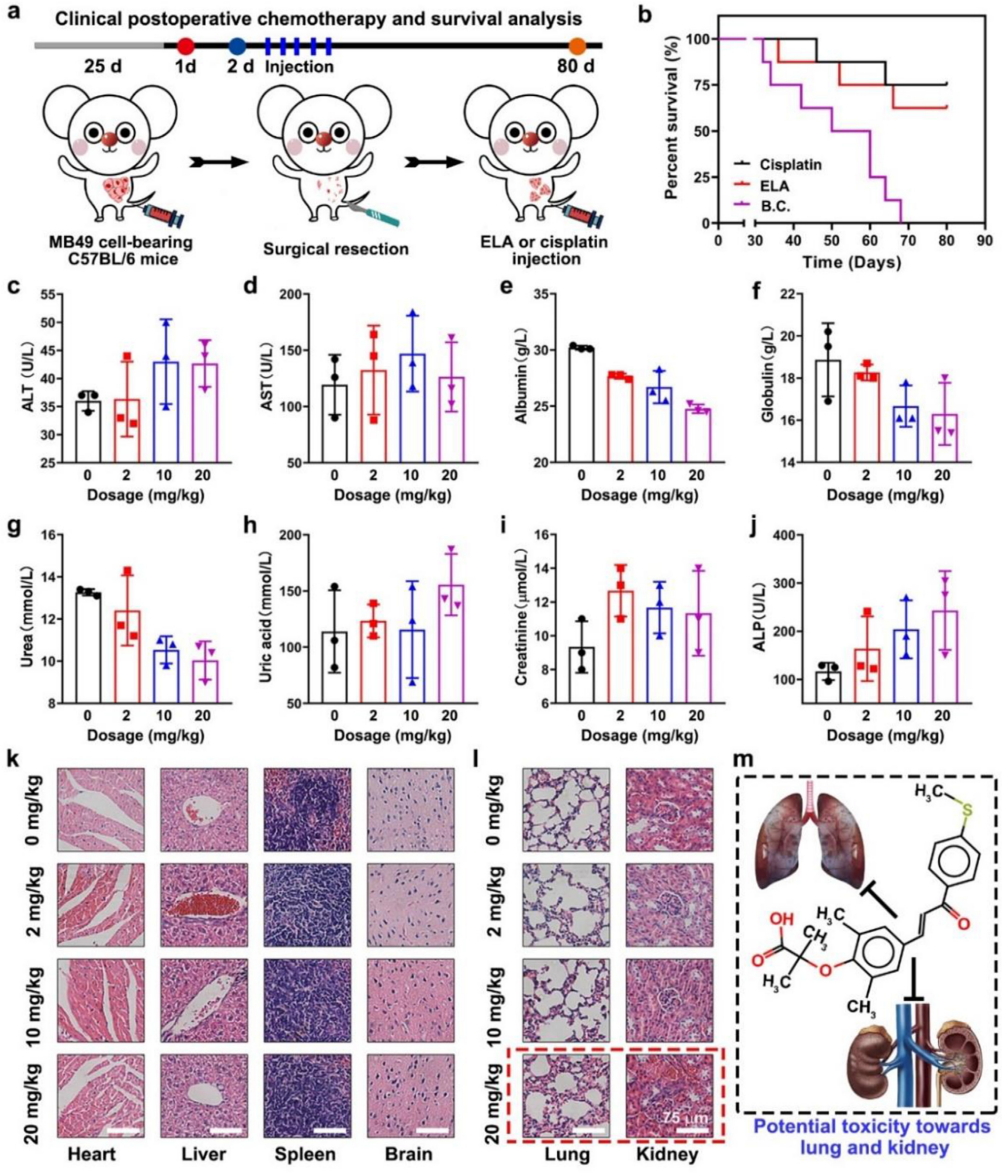
**ELA was biocompatible, and prolonged the survival in apost-operativerecurrence model.(**a) Schematic illustration of the construction of postoperative recurrence model, drug administration and follow up plan; (b) The survival curves of mice treated by PBS, ELA and cisplatin (n ​= ​8). ∗∗∗*P* < 0.001; (c–j) Results of biochemical tests, including alanine aminotransferase (ALT), aspartate aminotransferase (AST), albumin, globulin, urea, uric acid, creatinine and alkaline phosphatase (ALP) (n = 3); (k, l) HE staining images of the organs. Scale bar: 75 μm; (m) Schematic illustration of the organ toxicity of ELA. Values are expressed as the mean ± SD.

In this study, ELA treatment effectively inhibited the tumorigenesis of NMIBC without causing any obvious organ toxicity. The antitumor effect of ELA was validated in an immunodeficient tumor-bearing nude mouse model. However, immunodeficient animal models cannot truly recapitulate the complex characteristics of tumors, such as recurrence and metastasis. Herein, as shown in Fig. 5a, we constructed an immunocompetent tumor-bearing model by transplanting rat bladder cancer cells (MB49) into normal C57BL/6 mice. After that, 90% of the neo-tumor tissue was resected to simulate clinical surgical treatment. ELA and cisplatin were used for postoperative chemotherapy. Due to the effects of a series of factors *in vivo* and *in vitro*, residual tumors grow and eventually lead to the death of animals. The survival curves of animals subjected to different treatments are shown in Fig. 5b. Compared with that of the B.C. group, the survival time of the ELA group was significantly greater (*P* < 0.001). Our results suggested that ELA could prevent postoperative recurrence of bladder cancer. The effect of ELA at a dose of 2 mg/kg was similar to that of cisplatin at a dose of 10 mg/kg.

The biosafety of ELA was further evaluated by a series of biochemical and pathological tests. Our previous results indicated that 2 mg/kg of ELA could inhibit tumor progression and recurrence while triggering no obvious toxicity. However, whether a higher dose of ELA is potentially toxic remains to be determined. Herein, ELA working solution was injected into normal C57BL/6 mice. A series of biochemical indices were evaluated by an automatic biochemical analyzer. The results are shown in Fig. 5c–j and Table S2. With increasing dose of ELA, the ALT, AST, uric acid, creatinine, and ALP levels increased, while the albumin, globulin, and urea levels decreased. Fortunately, all of the indices were still within the normal range.

HE-stained images of the organs are shown in Fig. 5k-l, and Masson's trichrome staining images are shown in Fig. S7. The heart, liver, spleen, and brain in each group exhibited normal histological morphology. However, the glomerulus of the 20 mg/kg group exhibited obvious atrophy and necrosis. Moreover, there was obvious hyperemia and edema in the lung tissue of the 20 mg/kg group. As shown in Fig. 5m and 20 mg/kg ELA could damage the glomerulus, leading to leakage of water and sodium, which eventually increased the burden on the lung. The other groups, which were treated with an ELA dose of less than 20 mg/kg, showed no obvious pathological changes. Thus, we concluded that an ELA concentration up to 10 mg/kg was relatively safe.

### 2.3 Validation of ELA treatment by a humanized CRC model

**Fig. 6:**
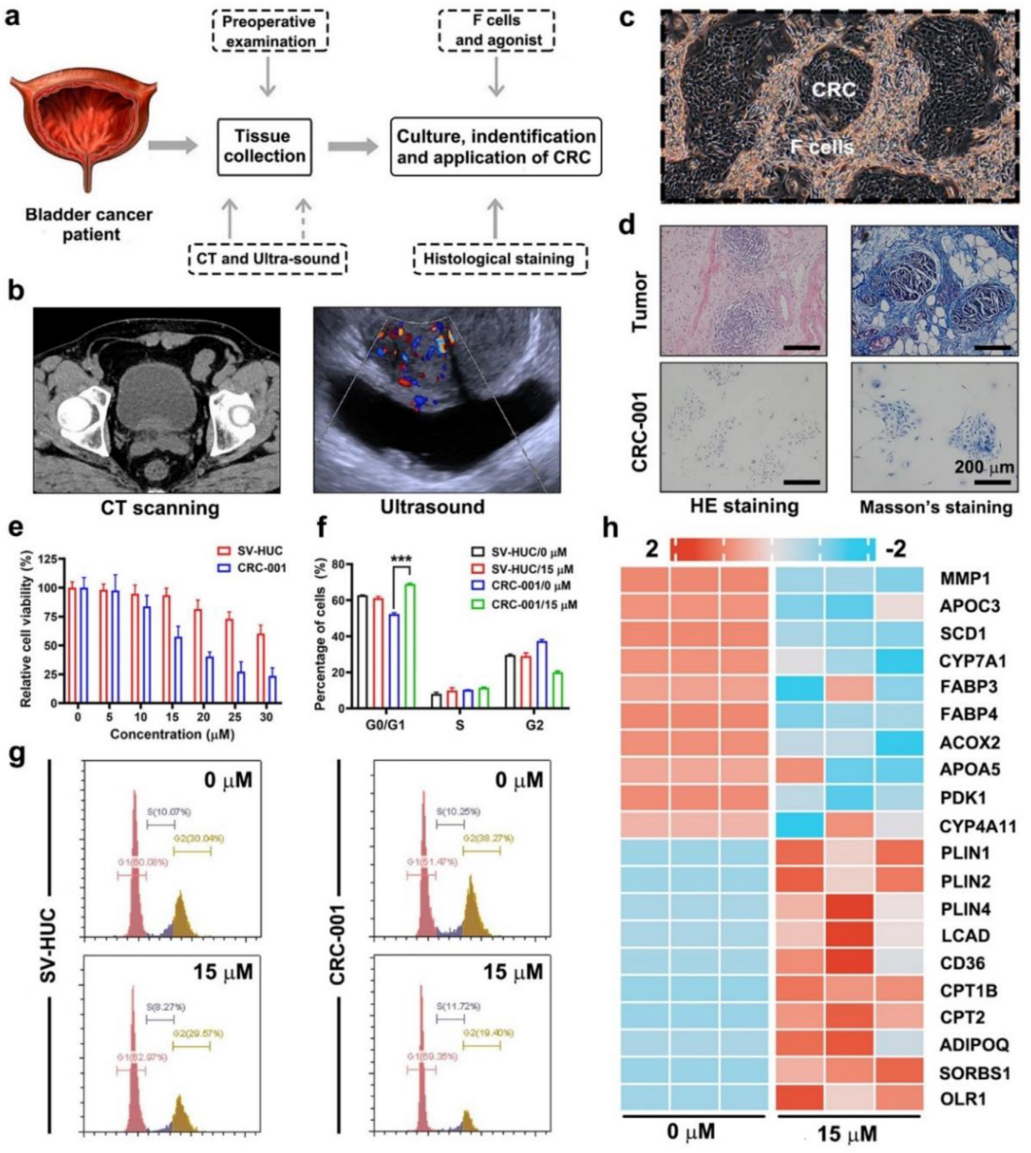
**Antitumor effect, tumor specificity, and mechanism of ELA treatment was validated by a humanized CRC model.** (a) Schematic illustration of the preparation of CRC; (b) Preoperative examinations of tissue donor from a muscle invasive bladder cancer patient; (c) Micro-images of the CRC; (d) Histological images of CRC and the parental tumor tissue. Scale bar: 200 μm; (e) The relatively viability of CRC-001 and SV-HUC (n ​= ​6); (f, g) Cell cycle analysis and the quantitative results (n ​= ​3); (h) Hot map of the hub genes of ELA-treated CRC in the PPAR pathway. Values are expressed as the mean ​± ​SD, ∗∗∗*P* < 0.001.

A humanized CRC model was constructed according to previous methods (Liu et al., 2017). A schematic illustration of CRC model construction is shown in Fig. 6a. A bladder cancer patient was examined via CT and ultrasound. After that, laparoscopic radical resection (LRS) of bladder cancer was performed, and fresh bladder cancer tissue was used for cell culture and histological identification.

The preoperative images are shown in Fig. 6b. The patient included in this study was diagnosed with muscle invasive bladder cancer, which is responsive to LRS treatment. As shown in Fig. 6c, the obtained CRCs grew in clusters with the help of feeder cells. This phenomenon was highly similar to that observed for tumor lesions *in vivo*. The HE and Masson's trichrome staining images are shown in Fig. 6d. The nuclei of the CRCs were basophilic and stained dark blue with hematoxylin. The cytoplasm of the CRCs was weakly eosinophilic and lightly stained with eosin. The histological characteristics of the CRCs were more similar to those of the tumor tissue than to those of the surrounding normal bladder tissue. The CRCs contained less collagen, indicating a highly proliferative state. Thus, we concluded that the CRCs was successfully cultured. The obtained CRC line was named CRC-001 for further study.

The antitumor effect of ELA was validated using a humanized CRC model. Normal human bladder epithelial cells (SV-HUC) served as the control. As shown in Fig. 6e, ELA was applied to SV-HUC and CRC-001 cells for 48 h. As the ELA concentration increased, the viability of both cell lines decreased gradually. CRC-001 cells were more sensitive to ELA treatment than SV-HUC cells. This result indicated that ELA treatment has certain tumor cell specificity, which is beneficial for cancer patients. We also used two independent groups, a blank control group (0 μM) and an ELA group (15 μM), for flow cytometry (Fig. 6f–g). The percentage of SV-HUC cells in the G0/G1 phase was 62.77% ± 0.17% in the 0 μM group and 61.16% ± 0.95% in the 15 μM group; no significant difference was observed (*P* > 0.05). Moreover, the percentage of CRC-001 cells in the G0/G1 phase was 52.28% ± 0.82% in the 0 μM group and 68.93% ± 0.39% in the 15 μM group, and a significant difference was observed (*P* < 0.001). These results further confirmed the antitumor effect and tumor specificity of ELA treatment.

ELA is the first coagonist of PPAR-α/δ. To determine the mechanism of ELA treatment in the CRC model, a series of hub genes in the PPAR pathway were analyzed via qRT‒PCR. A heatmap is shown in Fig. 6h, and the top 10 upregulated genes and the top 10 downregulated genes are listed. A series of biological processes, including lipid metabolism (SCD1, CYP7A1, ACOX2, LCAD, and CPT1B), gluconeogenesis (PDK1 and SORBS1), and fatty acid transport (FABP3, FABP4, and APOP5), are involved in ELA treatment. These results were consistent with our hypothesis of starvation therapy.

## 3 Discussion

Chemotherapy is one of the main treatment options for cancer. With the development of medical technology, the shortcomings of chemotherapy, such as insufficient effectiveness and biosafety, have become increasingly prominent (Wu et al., 2014; Zheng et al., 2023; Zhou et al., 2017). Traditional chemotherapeutics are cytotoxic and can cause serious side effects (Qiao et al., 2024; Yu et al., 2023). In this context, the screening of advanced chemotherapeutics is urgently needed. Previous studies have revealed that tumor cells are in a high metabolic state and that the energy demand of tumor cells was significantly greater than that of normal cells. Inspired by weight loss strategies, suppressing tumor cell energy metabolism may have antitumor potential. ELA is a dual inhibitor of glucose metabolism and lipid metabolism (Tsai et al., 2019, pp. 1–14). In this work, we established a strategy for treating NMIBC with ST and preliminarily evaluate the feasibility of ELA as a first-line treatment for NMIBC.

We found that 20 μM ELA could effectively inhibit the proliferation and migration of bladder cancer cells in an immortalized cell model. The mechanisms may include cell cycle arrest and increased intracellular ROS production. The results of *in vitro* experiments alone are not enough to support the whole process of drug transformation. In this work, we first subcutaneously transplanted T24 cells into BALB/C nude mice to construct an immunodeficient tumor-bearing model. We found that 2 mg/kg ELA could effectively inhibit the progression of primary tumors, and the underlying mechanism might be related to inhibiting cell proliferation and inducing apoptosis. Moreover, we constructed a recurrent tumor-bearing model in which mouse-derived MB49 cells were transplanted into normal C57BL/6 mice subcutaneously, surgical resection was simulated, and the tumors subsequently recurred *in situ*. These model mice have normal immune function, and the model itself more closely recapitulates clinical scenarios than other models (Giri et al., 2020; Simon, 2010). We found that ELA improved the survival time of treated animals. ELA was found to be relatively biosafe, although it was toxic to the lung and kidney at a dose 10 times higher than the dose used for drug administration (20 mg/kg).

Compared with animal experimental results, humanized research data are more convincing (Chen et al., 2020). There are few humanized research models of NMIBC. Based on our previous findings, our group successfully constructed a bladder cancer CRC line. Under the existing technical conditions, CRC has the maximum similarity with the parent tumor tissue (Wang et al., 2020; Yilmazer et al., 2015). We found that ELA inhibited the proliferation of CRCs more robustly than that of normal bladder epithelial cells. This result confirmed that ST using ELA had certain tumor specificity. In our mechanistic research, we preliminarily screened the molecular targets of ELA treatment. A series of metabolic processes were involved, which was consistent with our hypothesis about ST.

In this study, an immortalized cell model, an immunodeficient mouse model, an immunocompetent mouse model and a humanized CRC model were constructed to investigate the antitumor effect and biosafety of ELA from multiple dimensions. Notably, ELA can be used as a general drug for ST, but its application scope is not limited to NMIBC. We believe that ELA may have enough potential to be tested in clinical trials. This study has several limitations, such as the lack of in-depth mechanistic research and the small sample size of the humanized cell model; these limitations need further improvement. A series of preclinical research models were successfully constructed and are expected to be condensed into a methodology system that can be referenced and further used in future drug transformation research.

## 4 Methods

### 4.1 Materials

ELA (cat#: 923978-27-2) was purchased from Top-Science Co., Ltd. (Shanghai, China). Human bladder cancer cells (UMUC3 and T24), human bladder epithelial cells (SV-HUC) and rat bladder cancer cells (MB49) were kindly obtained from the National Collection of Authenticated Cell Cultures, Chinese Academy of Sciences (Shanghai, China). All cells were recently subjected to short tandem repeat (STR) certification by a third party. DMEM high glucose medium, RPMI-1640 basal medium, fetal bovine serum, antibiotics, phosphate-buffered saline (PBS), and trypsin were obtained from Gibco Co., Ltd. (Shanghai, China). A cell cycle staining kit (cat#: CCS012) was purchased from Multi-Science Co., Ltd. (Hangzhou, China). The ROS staining kits (cat#: 200-664-3) were purchased from Sigma-Aldrich Co., Ltd. The catalase (CAT, cat#: A007-1-1) and malonaldehyde (MDA, cat#: A003-4-1) kits were purchased from the Jian-Cheng Bioengineering Institute (Nanjing, China). N-acetylcysteine (NAC, cat#: 616-91-1) was purchased from Proteintech Group Co., Ltd. (Wuhan, China). The PPARα/δ target siRNAs were synthesized and purchased from GenePharma Co., Ltd. (Shanghai, China). The PPARα siRNA sequences was as follows: 5'-GCAGAAAUUCUUACCUGUGAATT-3'. The PPARδ siRNA sequences was as follows: 5'-AGAAGGCCCGCAGCAUCCUTT-3'. Lipofectamine 3000 (cat#: L3000015) was purchased from ThermoFisher Scientific Co., Ltd. (Shanghai, China). Primary antibody, including PNCA (cat#: 10205-2-AP), cyclin D1 (cat#: 26939-1-AP) and β-actin (cat#: 81115-1-RR) was purchased from Proteintech Group Co., Ltd. (Wuhan, China). Other chemical and biological reagents were used without purification.

### 4.2 Antitumor evaluations in an immortalized cell model

#### 4.2.1 MTT assay

An MTT assay was performed to evaluate the proliferation ability of ELA-treated cells, after which the appropriate concentration was determined for further study (Wang et al., 2021). Briefly, T24 and UMUC3 cells were seeded into 96-well tissue culture plates (TCPs) at a density of 3 × 10^3^ cells/well. After 24 h of incubation, all the medium was removed and replaced with 200 μL of fresh culture medium containing different doses of ELA. At regular time intervals, 20 μL of MTT agent was added to each well, and the mixture was incubated for another 4 h. After discarding the supernatant, the formazan crystals from each sample were dissolved in 150 μL of dimethyl sulfoxide. The absorbance at a wavelength of 490 nm was measured using a microplate reader (Spectra Max M2, Thermo Fisher, USA).

According to the manufacturer's protocols, PPARα/δ-siRNAs were transfected into T24 and UMUC3 cells using Lipofectamine 3000. After 48 h of incubation, the mRNA expression of PPARα/δ was measured via qRT‒PCR. The proliferation curves of ELA-treated cells with or without PPARα/δ silencing were recorded by MTT assay.

An MTT assay was also performed to evaluate whether NAC can counteract the viability of ELA-treated cells. T24 and UMUC3 cells were seeded into 96-well plates with a density of 3 × 10^3^ cells per well. After 24 h of incubation, all the medium was removed and replaced with 200 μL of fresh culture medium containing PBS, 20 μM ELA, or 20 μM ELA+10 μM NAC. The proliferation curves of the cells were recorded.

#### 4.2.2 Wound healing assay

The cells were seeded into 6-well plates and cultured until the cell confluence reached 95%. The cell monolayer was scratched with a 200 μL micropipette tip and then rinsed with precooled PBS three times to remove the unattached cells. Then, 2.5 mL of hypotrophic medium containing 2% FBS and 5 or 20 μM ELA was added to each well. After 24 h of incubation, the cell monolayer was observed and photographed using an inverted fluorescence microscope (IX73, OLYMPUS, Japan). The horizontal distance between the wound gaps was measured using ImageJ software. The migration ability of ELA-treated cells was further evaluated by a Transwell chamber assay, and the experimental methods can be found in the supplementary information.

#### 4.2.3 Transwell chamber assay

The cells were pretreated with 5 μM or 20 μM ELA for 24 h and then collected in serum-free medium at a density of 2.0 × 10^5^ per milliliter. Two hundred microliters of cell suspension was added to the upper layer of the Transwell chamber, and 700 μL of complete medium was added to the lower layer. After incubating at 37 °C for 24 h, the cells were fixed with 4% paraformaldehyde solution for 10 min, followed by staining with 0.1% crystal violet solution for another 30 min. The nonmigrated cells in the upper layer were carefully removed using cotton swabs. The migrated cells were then photographed using an inverted phase contrast microscope. At least five random fields were captured for statistical analysis.

#### 4.2.4 Flow cytometric analysis of the cell cycle

The cells were treated with ELA for 48 h. The obtained cells were rinsed with PBS three times and then stained with 1 mL of DNA staining solution and 10 μL of permeabilization solution in the dark for 30 min. The distribution of cells in different phases of the cell cycle was determined using a flow cytometer (Cytoflex, Beckman, China). For each group, 1 × 10^4^ cells were included for quantitative analysis. At least three independent samples were analyzed.

#### 4.2.5 Flow cytometry for ROS analysis

The ELA-treated cells were rinsed with PBS three times and then stained with 10 μM 2′,7′-dichlorodihydrofluorescein diacetate in the dark for 30 min. Afterward, the cells were rinsed with PBS three times to remove residual dye. The intracellular ROS level was assessed using a flow cytometer.

#### 4.2.6 Flow cytometry for cell apoptosis

The ELA-treated cells were rinsed with PBS three times, after which 2 μL of Annexin V-FITC and 4 μL of propidium iodide (PI) were added for staining. The mixture was incubated at room temperature in the dark for 15 min. After that, the cells were rinsed with PBS three times to remove the residual dye. Apoptosis was detected using a flow cytometer.

#### 4.2.7 Biochemical tests

The levels of ROS metabolites and the activity of ROS catalytic enzymes were determined to further evaluate intracellular ROS production. The cells were treated with ELA for 48 h and then lysed using a freeze‒thaw cycling technique. The cell lysate was diluted twice with PBS and then stored at 4 °C. Based on previous reports (Nascimento et al., 2021), CAT activity and MDA levels were evaluated. All of the tests were carried out according to the manufacturer's standard protocols. At least three independent samples were used for statistical analysis.

#### 4.2.8 Total RNA isolation and qRT‒PCR

Total RNA was isolated from ELA-treated cells using an RNeasy Mini Kit (Qiagen, Germany). Reverse transcription was carried out to prepare cDNA according to the instructions of the ReverTrace qRT‒PCR Kit (Bio-Rad, USA). The qRT‒PCR reaction included 3 μL of primer mixture, 4.5 μL of cDNA and 7.5 μL of iQTM SYBR® Green Supermix (Bio-Rad, USA). The primer sequences for qRT‒PCR are listed in Table S1. The cycle threshold (Ct) was calculated compared to that of β-actin.

#### 4.2.9 Western blot analysis

A cell lysis solution was used to disrupt the cells after ELA treatment for total protein extraction. The protein concentration was determined using the BSA method. Subsequently, the proteins were separated via SDS-PAGE and subsequently transferred to a polyvinylidene fluoride membrane. After antibody incubation, enhanced chemiluminescence (ECL) staining was carried out, and protein bands were visualized with Biomax MR films (Kodak, Rochester, NY).

### 4.3 Xenograft animal model

This work was approved by the Animal Care and Welfare Committee of Wuhan University (No: 201902) and carried out in accordance with the “Guidelines and Regulations for the Use and Care of Animals” of the Review Board of Hubei Medical Laboratory Animal Center. Immunodeficient BALB/c nude mice were purchased from Shulaibao Biotech. Co., Ltd. (Wuhan, China) and subsequently raised in a specific pathogen-free (SPF) animal center for 7 days to relieve further stress.

Healthy T24 cells were rinsed with PBS three times to remove organic solutes. The cell density was adjusted to 1 × 10^7^ per milliliter. The animals were safely fixed, and their right upper armpits were disinfected using 75% alcohol solution. A total of 200 μL of cell suspension was injected, followed by compression using a cotton swab for at least 30 s. Afterward, the animals were transferred to the SPF animal center and conventionally fed. We recorded the dietary intake, daily activities and behavior of the treated animals.

Fifteen days after cell transplantation, the neotumour tissue in each mouse was visible to the naked eye. Ten mice were randomly divided into 2 groups, the blank control group and the ELA group. ELA was first dissolved in alcohol to prepare a 10 mg/mL stock solution and then diluted with PBS to obtain a 2 mg/mL working solution. The working solution was injected directly into the tumor tissue at a dose of 2 mg/kg. For the blank control group, the animals were treated with an equal volume of PBS containing alcohol. Drug administration was repeated every three days.

At regular time intervals, the major diameter (Dmax) and minor diameter (Dmin) of the neotumour tissue were measured using a Vernier caliper. The volume of the tumor was calculated as follows:

$$\text{Volume}\;\text{of}\;\text{tumor}\;\left(\text{Vt}\right)=0.5\times \text{Dmax}\times {\text{Dmin}}^{2}$$

On Day 30, all the animals were sacrificed. The tumor tissues were anatomically separated, photographed and weighed. The organ samples, including heart, liver, spleen, lung, kidney and brain, were also resected. All tissue samples were fixed with 4% paraformaldehyde solution for 72 h and then subjected to histopathological analysis. In this work, H&E staining, Masson staining, Ki67 immunohistochemical (IHC) staining, and TdT-mediated dUTP nick end labeling (TUNEL) were performed by a third-party testing organization (Baifeier, Wuhan, China). All of the experiments were performed according to standard protocols.

### 4.4 Recurrent tumor growth model

Immunocompetent C57BL/6 mice were purchased from Shulaibao Biotech. Co., Ltd. (Wuhan, China). Rat bladder cancer cells (MB49) were transplanted into these animals to construct an immunocompetent tumor-bearing model. To simulate clinical surgical treatment, 90% of the tumor tissue from each mouse was resected. After that, the animals were randomly divided into 3 groups, with 8 animals in each group. For the ELA-treated group, 2 mg/kg ELA was injected into the resection site to prevent tumor recurrence. For the blank control group, the animals were injected with an equal amount of PBS. Additionally, a positive control group was also included. Cisplatin was injected at a dose of 10 mg/kg. Drug administration was carried out 5 times every three days. After that, the animals were conventionally fed, allowing tumor recurrence. The survival curves of these animals were recorded.

### 4.5 Biosafety evaluation *in vivo*

Normal C57BL/6 mice were randomly divided into 4 groups. These animals were injected intraperitoneally with ELA at doses of 0, 2, 10 and 20 mg/kg. Drug administration was carried out 5 times every three days. After that, the animals were anesthetized by isoflurane inhalation, and fresh whole blood was collected by heart puncture. Blood samples were centrifuged at 3000 rpm for 10 min to remove blood cells. Blood biochemical tests were performed at the Clinical Laboratory, Hubei Provincial Hospital of Traditional Chinese Medicine. Three independent samples were used for statistical analysis. Moreover, organ samples, including heart, liver, spleen, lung, kidney and brain tissue, were resected and fixed using 4% PFA solution. Hematoxylin and eosin (HE) staining and Masson staining were performed to evaluate organ toxicity.

### 4.6 Validation using a humanized CRC model

This work was approved by the Ethics Committee of Zhongnan Hospital, Wuhan University (No: 2020102). Fresh tissue was collected from a newly diagnosed muscle-invasive bladder cancer (MIBC) patient with informed consent.

A conditional reprogramming technique was introduced to prepare the humanized cells. A bladder cancer primary cell isolation kit (YTB-ISO-01) and feeder cells (YTB-IR-L201) were purchased from Yongtai Biotech. Co., Ltd. (Shenzhen, China). According to the general protocols, fresh tissue was digested into single cells and then cocultured with feeder cells and primary culture medium containing 10% fetal bovine serum. The feeder cells and culture media were replaced every three days. After 15–20 days of culture, the humanized BCa cells were successfully prepared for further study.

The cells were treated with ELA for 48 h, after which cell viability was determined via the MTT assay. Normal human bladder epithelial cells (SV-HUC-1) served as the control group. Furthermore, the cell cycle distribution was investigated by flow cytometry. The expression of several hub genes in the PPAR signaling pathway was measured by qRT‒PCR. The sequences of primers used are listed in Supplementary Table S1. The cycle threshold (Ct) was calculated compared to that of GAPDH.

### 4.7 Ethics statement

All experiments were permitted by the Precision Medicine Laboratory, Zhongnan Hospital of Wuhan University. The use of clinical samples was approved by the Ethical Committee of Zhongnan Hospital of Wuhan University, and written informed consent was obtained from all the patients. All animal experiments were approved by the Experimental Animal Welfare and Ethics Committee, Zhongnan Hospital of Wuhan University.

### 4.8 Statistical analysis

The Graph-Pad Prism 7.0 software was used for statistical analysis. Histological images were analyzed by Image-J v1.45 software. The quantitative data were expressed as mean ± standard deviation. The statistical method was one-way ANOVA. At least three independent samples were used for statistical analysis. *P* < 0.05 indicated significant difference.

## Author contributions

WW: data collection, statistical analysis, interpretation of results and manuscript writing. DS: data acquisition, statistical analysis and interpretation of results. GW and XM: data analysis and processing. YW: revision of the manuscript. XW and ZW: review, revision of the manuscript; material support and study supervision. All authors read and approved the final manuscript.

## Declaration of competing interest

The authors declare that they have no known competing financial interests or personal relationships that could have appeared to influence the work reported in this paper.
