# Supplementary material for: Elafibranor emerged as a potential chemotherapeutic drug for non-muscle invasive bladder cancer
Source: Cell Insight. 2024 Jan 29;3(1):100149. doi: 10.1016/j.cellin.2024.100149 (PMC10840351; doi:10.1016/j.cellin.2024.100149)
Supplement: Multimedia component 1 [file mmc1.docx]

**Elafibranor emerged as a potential chemotherapeutic drug for non-muscle invasive bladder cancer**

Wang Wang^a,1^, Danni Shan^a,1^, Guanyi Wang^a^, Xiongmin Mao^a^, Wenjie You^b,***^, Xiaolong Wang^a,c,**^, Zijian Wang^a,d,*^

^a^ Department of Urology, Cancer Precision Diagnosis and Treatment and Translational Medicine Hubei Engineering Research Center, Zhongnan Hospital of Wuhan University, Wuhan 430071, China.

^b^ Orthopedic Hospital, The First Affiliated Hospital, Jiangxi Medical College, Nanchang University, Nanchang, 330006, China.

^c^ Lewis Katz School of Medicine, Temple University, Philadelphia, PA 19140, USA.

^d^ Department of Biomedical Engineering and Hubei Province Key Laboratory of Allergy and Immune Related Disease, Taikang Medical School (School of Basic Medicine Sciences), Wuhan University, Wuhan 430071, China.

^1^ These authors contributed equally to this work

* Corresponding authors.

** Corresponding authors.

*** Corresponding authors.

E-mail addresses: [youwenjie@ncu.edu.cn](mailto:youwenjie@ncu.edu.cn) (W. You); [nogardmd@whu.edu.cn](mailto:nogardmd@whu.edu.cn) (X. Wang); [Zijianwang@whu.edu.cn](mailto:Zijianwang@whu.edu.cn) (Z. Wang).

**Table S1.** Codes and sequences of primers used for qRT-PCR analysis

| **Codes** | **Species** | **Primer sequences (5’-3’)** | **Amplicon Size (bp)** | **Tm (℃)** |
| --- | --- | --- | --- | --- |
| β-Actin | Human | Forward: GATCCACATCTGCTGGAAG | 579 | 55.06 |
|  |  | Reverse: ATCTACGAGGGGTATGCC |  | 55.06 |
| MMP1 | Human | Forward: CTCTGGAGTAATGTCACACCTCT | 199 | 59.23 |
|  |  | Reverse: TGTTGGTCCACCTTTCATCTTC |  | 58.51 |
| APOC3 | Human | Forward: CCGCCAAGGATGCACTGAG | 100 | 61.11 |
|  |  | Reverse: CTCCAGTAGTCTTTCAGGGAACT |  | 59.16 |
| SCD1 | Human | Forward: GCCCCTCTACTTGGAAGACGA | 161 | 61.23 |
|  |  | Reverse: AAGTGATCCCATACAGGGCTC |  | 59.23 |
| CYP7A1 | Human | Forward: GCAATTTGGTGCCAATCCTCT | 133 | 59.45 |
|  |  | Reverse: GCACAACACCTTATGGTATGACA |  | 58.99 |
| FABP4 | Human | Forward: ACTGGGCCAGGAATTTGACG | 183 | 60.61 |
|  |  | Reverse: CTCGTGGAAGTGACGCCTT |  | 60.01 |
| ACOX2 | Human | Forward: GCACCCCGACATAGAGAGC | 99 | 59.93 |
|  |  | Reverse: CTGCGGAGTGCAGTGTTCT |  | 60.30 |
| APOA5 | Human | Forward: GCTGGTGGGCTGGAATTTG | 241 | 59.41 |
|  |  | Reverse: CTCGGCGTATGGGTGGAAG |  | 60.23 |
| PDK1 | Human | Forward: CTGTGATACGGATCAGAAACCG | 191 | 58.88 |
|  |  | Reverse: TCCACCAAACAATAAAGAGTGCT |  | 58.53 |
| CYP4A11 | Human | Forward: CCATCCCCATTGCACGACTT | 274 | 60.68 |
|  |  | Reverse: CAGGTAGACAAGCAGGTAGGG |  | 59.52 |
| FABP3 | Human | Forward: GGCACCTGGAAGCTAGTGG | 78 | 60.08 |
|  |  | Reverse: CTGCCTGGTAGCAAAACCC |  | 58.73 |
| PLIN1 | Human | Forward: TGTGCAATGCCTATGAGAAGG | 154 | 58.35  60.62 |
|  |  | Reverse: AGGGCGGGGATCTTTTCCT |  |  |
| LCAD | Human | Forward: AGGGGATCTGTACTCCGCAG | 190 | 60.76  58.99 |
|  |  | Reverse: CTCTGTCATTGCTATTGCACCA |  |  |
| CD36 | Human | Forward: AAGCCAGGTATTGCAGTTCTTT | 220 | 58.50  59.58 |
|  |  | Reverse: GCATTTGCTGATGTCTAGCACA |  |  |
| CPT1B | Human | Forward: GCGCCCCTTGTTGGATGAT | 112 | 60.75  61.22 |
|  |  | Reverse: CCACCATGACTTGAGCACCAG |  |  |
| ADIPOQ | Human | Forward: TGCTGGGAGCTGTTCTACTG | 248 | 59.39  59.80 |
|  |  | Reverse: TACTCCGGTTTCACCGATGTC |  |  |
| CPT2 | Human | Forward: CATACAAGCTACATTTCGGGACC | 183 | 59.44  60.83 |
|  |  | Reverse: AGCCCGGAGTGTCTTCAGAA |  |  |
| PLIN4 | Human | Forward: GGAGCTGCAACCTTCGGAAA | 311 | 60.89 |
|  |  | Reverse: GGACCACTCCCTTAGCCAC |  | 59.40 |
| PLIN2 | Human | Forward: TTGCAGTTGCCAATACCTATGC | 148 | 59.57 |
|  |  | Reverse: CCAGTCACAGTAGTCGTCACA |  | 59.39 |
| SORBS1 | Human | Forward: CACAATCGAGAACAGCAAAAACG  Reverse: ACCCGCCTACTGTCATCCTTT | 152 | 59.58  61.18 |
| OLR1 | Human | Forward: TTGCCTGGGATTAGTAGTGACC | 81 | 59.50 |
|  |  | Reverse: GCTTGCTCTTGTGTTAGGAGGT |  | 60.55 |
| PPARα | Human | Forward: ATGGTGGACACGGAAAGCC | 124 | 62.3 |
|  |  | Reverse: CGATGGATTGCGAAATCTCTTGG |  | 61.8 |
| PPARδ | Human | Forward: CAGGGCTGACTGCAAACGA | 191 | 62.5 |
|  |  | Reverse: CTGCCACAATGTCTCGATGTC |  | 61.0 |

**Table S2.** Results of plasma biochemical tests

|  | Unit | 0 mg/kg | 2 mg/kg | 10 mg/kg | 20 mg/kg |
| --- | --- | --- | --- | --- | --- |
| Cholinesterase | U/L | 4074 ± 229 | 3679 ± 319 | 3333 ± 120 | 3292 ± 137 |
| Total bile acid | μmol/L | 2 ± 1 | 2 ± 0 | 1.67 ± 0.58 | 1.67 ± 0.58 |
| Glucose | mmol/L | 9.7 ± 1.8 | 12.8 ± 0.6 | 10.0 ± 3.3 | 14.1 ± 1.8 |
| K | mmol/L | 4.98 ± 0.24 | 5.82 ± 0.04 | 6.07 ± 0.30 | 5.22 ± 1.41 |
| Na | mmol/L | 151.1 ± 1.9 | 149.7 ± 1.1 | 149.1 ± 2.4 | 147.2 ± 0.4 |
| Cl | mmol/L | 113.9 ± 1.9 | 113.0 ± 1.4 | 113.8 ± 2.0 | 110.2 ± 0.9 |
| Ca | mmol/L | 2.3 ± 0.1 | 2.4 ± 0.1 | 2.3 ± 0.1 | 2.4 ± 0.1 |
| P | mmol/L | 3.2 ± 0.9 | 4.0 ± 0.6 | 3.7 ± 0.8 | 2.8 ± 0.7 |
| CO_2_ | mmol/L | 16.7 ± 3.1 | 12.4 ± 3.1 | 12.3 ± 3.3 | 17.6 ± 2.5 |
| Cystatin-C | mg/L | 0.16 ± 0.03 | 0.20 ± 0.05 | 0.16 ± 0.02 | 0.15 ± 0.01 |


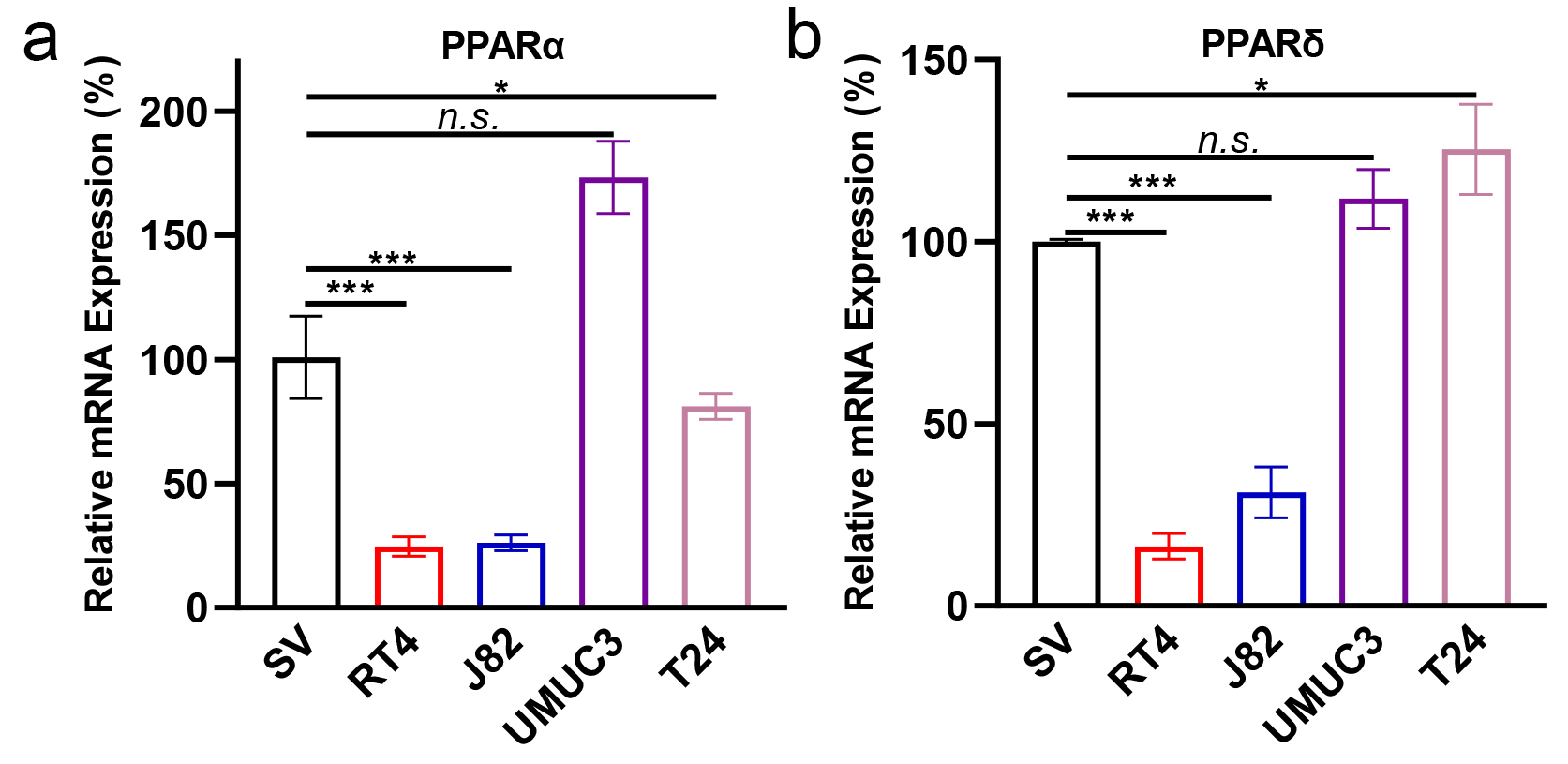


**Fig. S1**. (a) The relative mRNA expression of PPARα; (b) The relative mRNA expression of PPARδ. **P* < 0.05. ****P* < 0.001, *n.s.* indicated no significance.


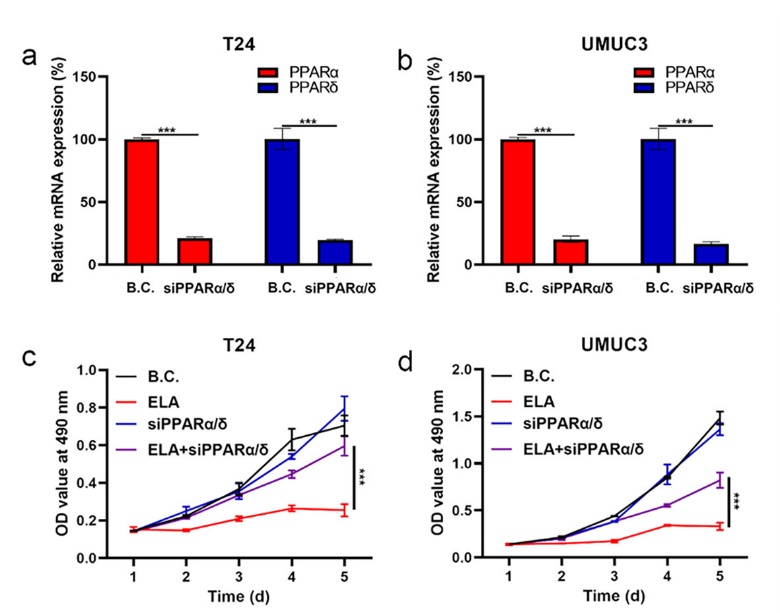


**Fig. S2**. (a-b) The PPARα/δ-siRNA silencing efficiency in T24 and UMUC3 cell lines (n = 3). (c-d) The proliferation curves of T24 and UMUC3 cells (n = 3). ****P* < 0.001.


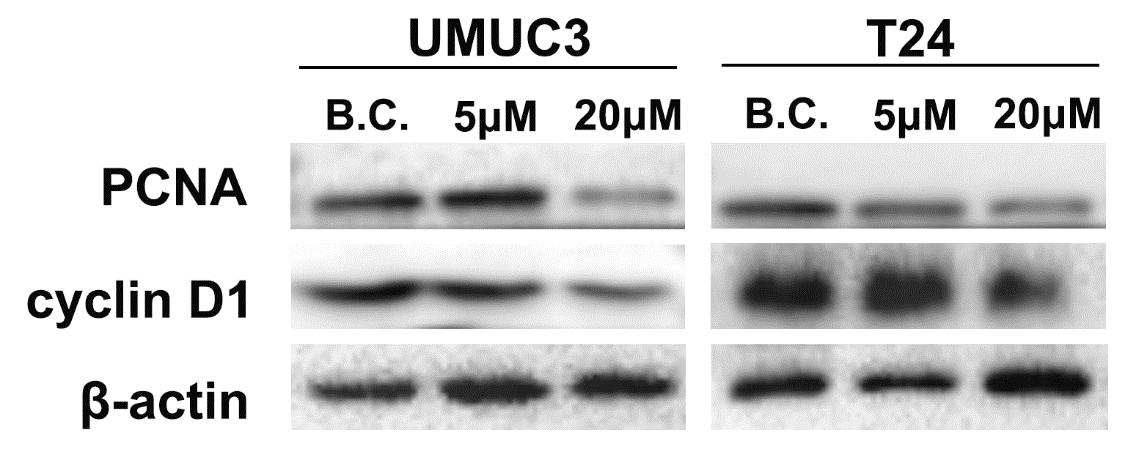


**Fig. S3**. Western blot analysis of PCNA, cyclin D1 after ELA treatment in two cell lines. β-actin was used as internal reference.


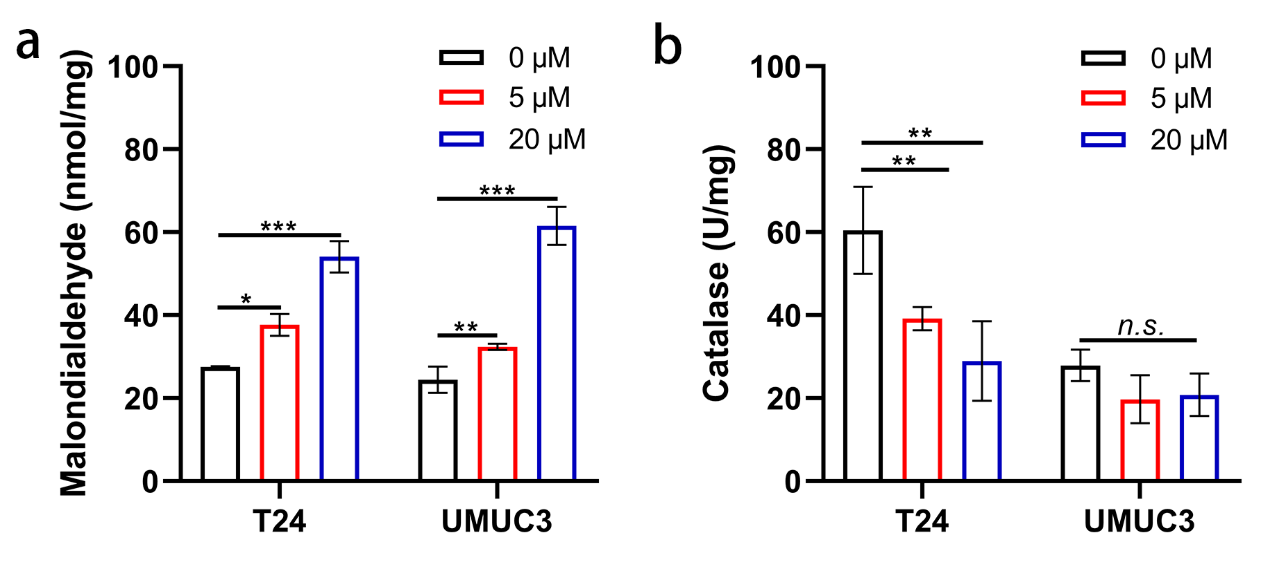


**Fig. S4**. (a) Intracellular content of MDA (n = 5); (b) Intracellular enzyme activity of CAT (n = 5). **P* < 0.05, **P < 0.01, ***P < 0.001, *n.s.* indicated no significance.

**
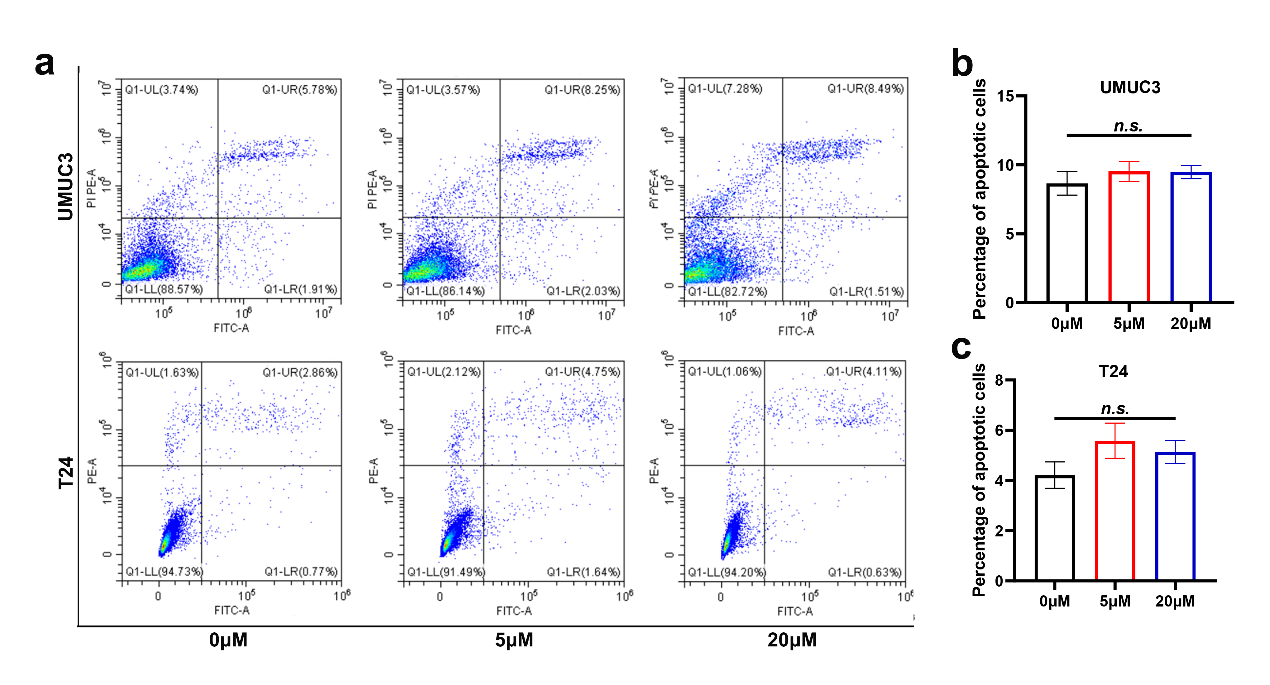
**

**Fig. S5**. (a) Cell apoptosis of T24 and UMUC3 cell was detected by flow cytometry. (b) Quantitative results of UMUC3 cell (n = 3); (c) Quantitative results of T24 cell (n = 3). N.S. indicated no significance.


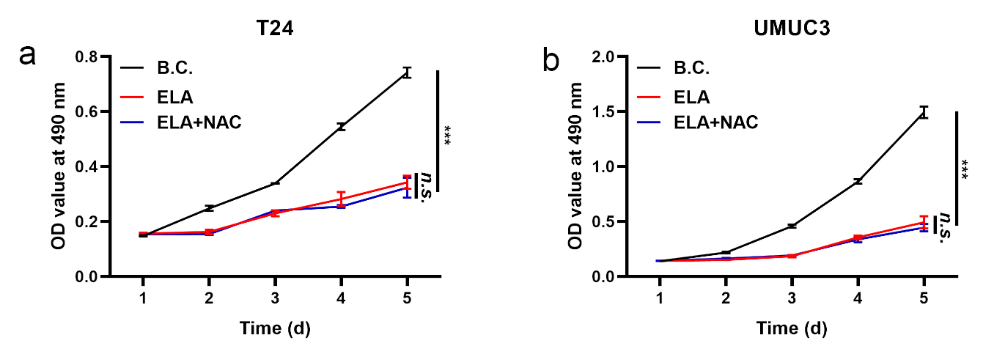


**Fig. S6.** (a-b) The proliferation curves of T24 and UMUC3 cell lines treated with PBS (B.C. group), 20 µM ELA (ELA group) and 20 µM ELA+10 µM NAC (ELA+NAC group). ****P* < 0.001, n.s. indicated no significance.

**
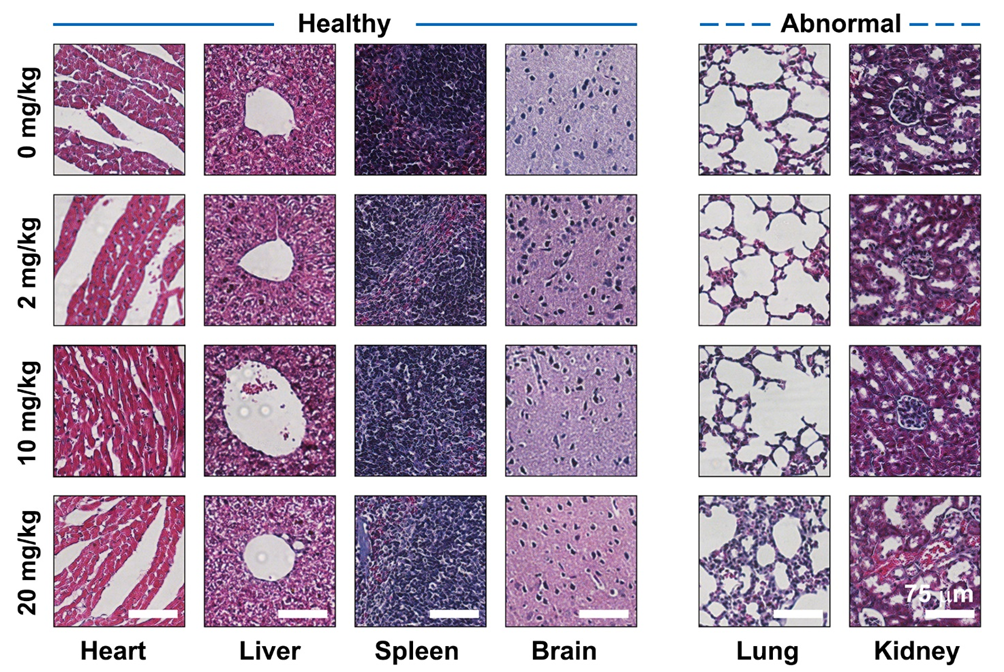
**

**Figure S7.** Healthy and abnormal Masson’s staining images of the organs from C57BL/6 mice. Scale bar: 75 µm.
